# Cross-sectional angle prediction of lipid-rich and calcified tissue on computed tomography angiography images

**DOI:** 10.1007/s11548-024-03086-2

**Published:** 2024-03-13

**Authors:** Xiaotong Zhang, Alexander Broersen, Hessam Sokooti, Anantharaman Ramasamy, Pieter Kitslaar, Ramya Parasa, Medeni Karaduman, Amear Souded Ali Jan Mohammed, Christos V. Bourantas, Jouke Dijkstra

**Affiliations:** 1 Division of Image Processing, Radiology, Leiden University Medical Center, Leiden, The Netherlands; 2 Cardiology, Barts Heart Centre, Barts Health NHS Trust, London, UK; 3 Medis Medical Imaging, Leiden, The Netherlands; 4 Centre for Cardiovascular Medicine and Devices, William Harvey Research Institute, Queen Mary University of London, London, UK; 5 Cardiology, Van Yuzuncu Yil University, Van, Turkey; 6 School of Engineering and Material Science, Queen Mary University of London, London, UK; 7 The Essex Cardiothoracic Centre, Basildon, UK

**Keywords:** Plaque detection, Spread-out view, CTA, 2.5D, Dense U-Net, Mask R-CNN

## Abstract

**Purpose:**

The assessment of vulnerable plaque characteristics and distribution is important to stratify cardiovascular risk in a patient. Computed tomography angiography (CTA) offers a promising alternative to invasive imaging but is limited by the fact that the range of Hounsfield units (HU) in lipid-rich areas overlaps with the HU range in fibrotic tissue and that the HU range of calcified plaques overlaps with the contrast within the contrast-filled lumen. This paper is to investigate whether lipid-rich and calcified plaques can be detected more accurately on cross-sectional CTA images using deep learning methodology.

**Methods:**

Two deep learning (DL) approaches are proposed, a 2.5D Dense U-Net and 2.5D Mask-RCNN, which separately perform the cross-sectional plaque detection in the Cartesian and polar domain. The spread-out view is used to evaluate and show the prediction result of the plaque regions. The accuracy and F1-score are calculated on a lesion level for the DL and conventional plaque detection methods.

**Results:**

For the lipid-rich plaques, the median and mean values of the F1-score calculated by the two proposed DL methods on 91 lesions were approximately 6 and 3 times higher than those of the conventional method. For the calcified plaques, the F1-score of the proposed methods was comparable to those of the conventional method. The median F1-score of the Dense U-Net-based method was 3% higher than that of the conventional method.

**Conclusion:**

The two methods proposed in this paper contribute to finer cross-sectional predictions of lipid-rich and calcified plaques compared to studies focusing only on longitudinal prediction. The angular prediction performance of the proposed methods outperforms the convincing conventional method for lipid-rich plaque and is comparable for calcified plaque.

## Introduction

Coronary artery disease (CAD) is one of the primary causes of death in the world. Recent studies have demonstrated that outcomes in patients with CAD depend not only on lesion severity but also on its composition. Lesion with a lipid-rich phenotype is at a higher risk to progress and causes major adverse cardiovascular events such as death or an acute coronary syndrome (ACS) [1–4]. In order to accurately diagnose the severity of CAD and characterize plaque phenotype, image processing techniques used for atherosclerotic plaque detection should have the capacity to describe plaque composition and location in as much detail as possible.

In general, invasive imaging modalities, such as intravascular ultrasound (IVUS) [5], optical coherence tomography (OCT) [6] and near-infrared spectroscopy (NIRS) [7], are considered as the gold standard for characterizing plaque composition which closely matches the histopathology of the plaque. However, these approaches are invasive, are associated with risk and can be used only in patients with symptomatic obstructive CAD. Non-invasive modalities such as computed tomography angiography (CTA) have been widely used for atherosclerotic plaque detection as they enable complete assessment of the coronary artery tree and can assess coronary artery pathology [8, 9]. However, due to the significant attenuation overlap between lipid-rich plaques and fibrotic or even the surrounding myocardial tissue, it is a challenging task for the cardiologist to distinguish lipid-rich plaques from normal tissue and mixed plaques from calcified plaques on CTA images solely based on the Hounsfield units (HU) range [10].

To avoid tedious manually crafted and complex feature extractions, many [3, 8, 11, 12] deep learning (DL)-based methods have been proposed to classify atherosclerotic plaques on IVUS, OCT and CTA images. For the CTA-based methods, Zreik et al. [11] proposed a method that has a recurrent convolutional neural network (CNN) architecture to classify calcified and non-calcified plaques and evaluate stenosis degree on multi-planar reformatted (MPR) images. Liu et al. [12] utilized the straightened MPR volume as the input of a 3D CNN and predicted calcified, non-calcified and mixed plaque regions on a 3D CT volume. However, both methods provide a coarse plaque range prediction on the axial direction of vessel instead of a detailed prediction on the cross-sectional view.

Considering the limitations of existing methods [3, 8, 11, 12] for atherosclerosis plaque classification and the need to accurately classify plaque phenotypes we introduce alternative DL approaches for the prediction of plaque types that were trained and tested using NIRS-IVUS pull-backs as the gold standard that were matched with CTA images. In the following part of the paper, we use the term *plaque* to represent the lipid-rich or calcified tissue.

Inspired by the classical architectures of the 2.5D U-Net [13], DenseNet [14] and the Mask R-CNN [15], two different methods for CT cross-sectional plaque classification are presented in this paper. This paper mainly contributes to three points. (1) The method is independent of the EEM detection. (2) Plaque angle prediction: this study focuses on the cross-sectional plaque distribution instead of longitudinal detection. (3) Refined visualization: the spread-out view from NIRS is used to evaluate and visualize the angle-wise plaque distribution.

## Materials

### Data acquisition

We analyzed 176 cardiac vessels from 64 patients who were prospectively recruited in the study, which aimed to examine the efficacy of CTA in assessing plaque pathology and physiology using NIRS-IVUS imaging as reference standard. In total 64 patients with obstructive CAD and typical angina symptoms were recruited and underwent CTA before being listed for percutaneous coronary intervention or functional assessment—depending on the clinical indication—and NIRS-IVUS imaging. The CTA was obtained by a dual-source CT scanner (Somatom Force, Siemens Healthineers, Forchheim, Germany). CTA images were reconstructed by highest strength model-based iterative reconstruction (ADMIRE 5) with a slice thickness of 0.50 mm and increments of 0.30 mm. NIRS-IVUS was performed in the major epicardial vessels and their large side branches with a diameter of more than 2 mm by a 2.4F MakotoTM NIRS-IVUS 35-65MHz Imaging System (Infraredx, Burlington, USA) that was withdrawn at a constant pull back speed 0.5 mm/s.

### Ground truth

**Fig. 1:**
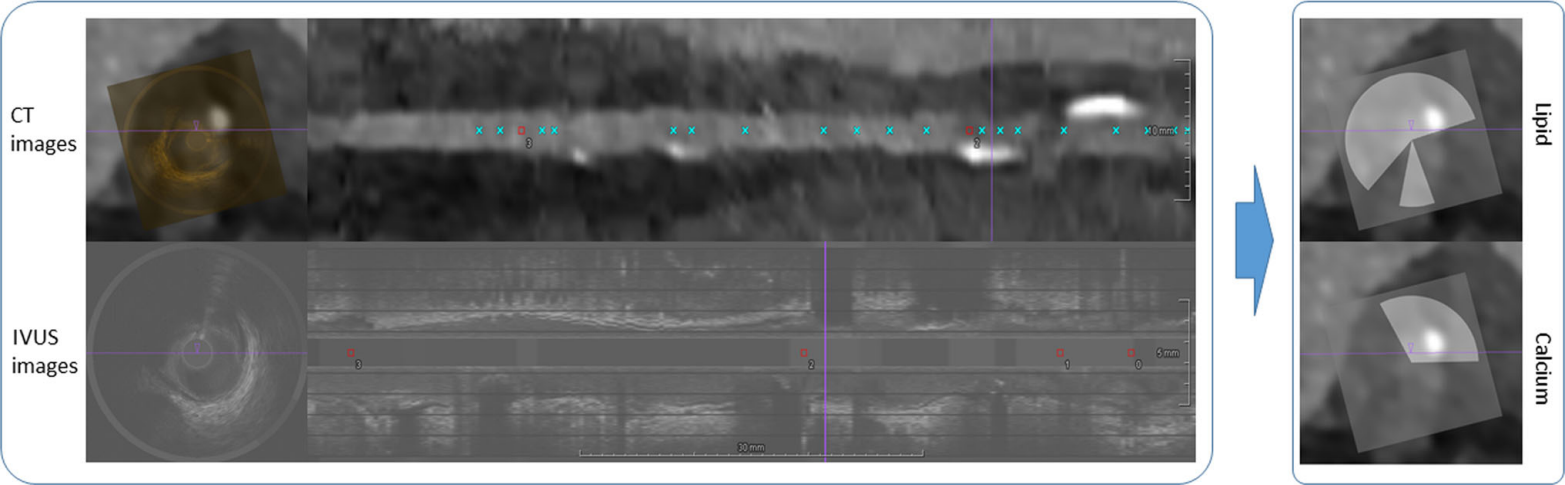
Vessel registration process to map IVUS images onto CT images

To train and test the DL methods using NIRS-IVUS images we co-registered these data with the CTA images; the registration process [16] shown in Fig. 1 and the plaque estimations of NIRS-IVUS were matched to the CT domain. The accuracy of the registration is closely related to the quality of the training labels, which affect the model performance directly. Several anatomical information that is seen in both modalities was used for this process including the origin of the side branches, location of the plaque and the presence of large calcific spots that are clearly visible in both datasets.

The plaque locations on IVUS were represented as pie-shaped labels as shown in Fig. 1, since NIRS only provides the angles with a high lipid plaque probability and calcium plaques block the ultrasound signal so the actual depth of calcium is not defined precisely.

**Fig. 2:**
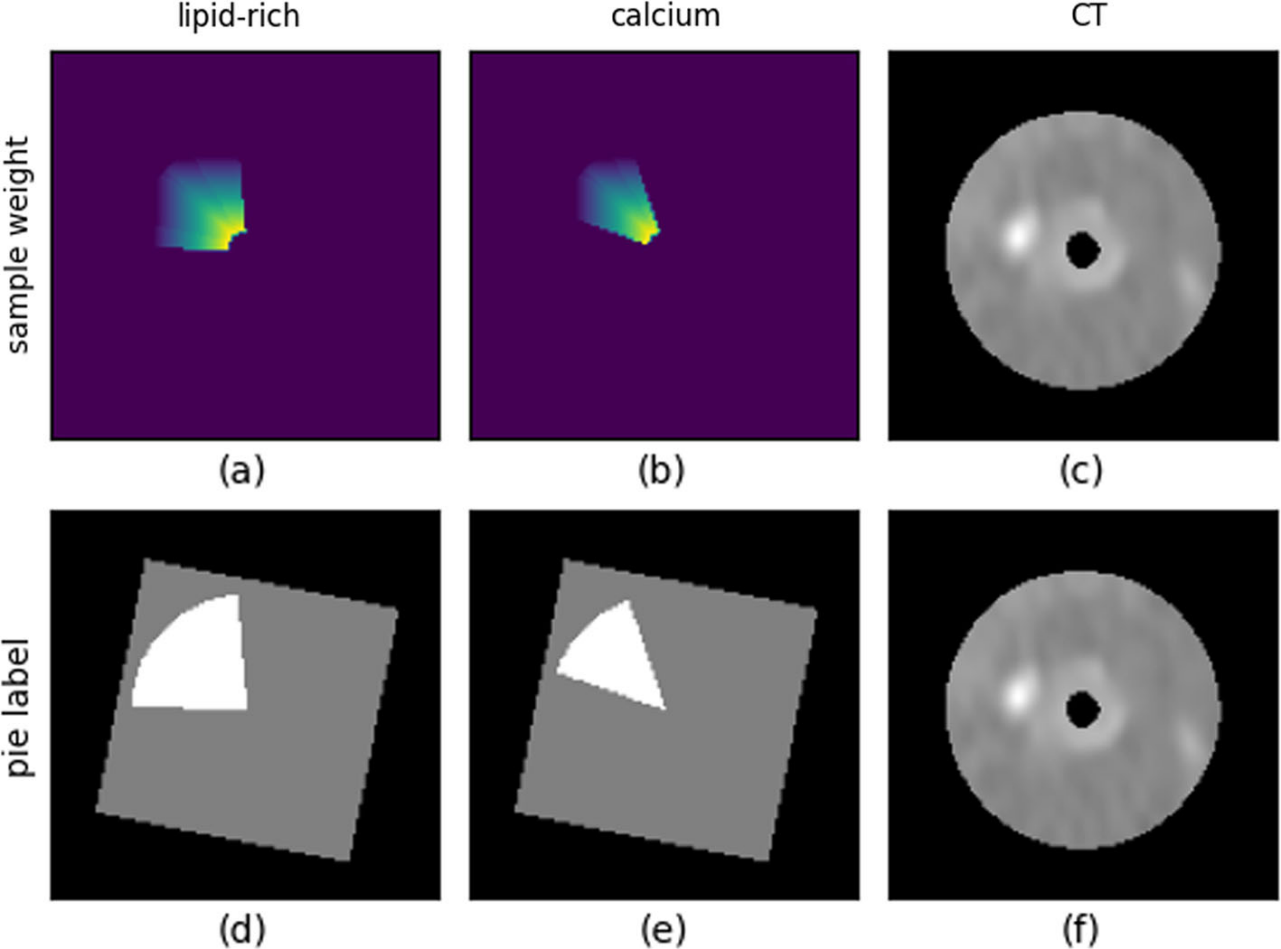
Binary labels and sample weights used for 2.5D Dense U-Net. **a**, **b**: pixel-wise gradient sample weights for lipid-rich plaques and calcified plaques; **d**, **e**: binary pie-shaped labels for lipid-rich and calcified plaques; **c**, **f** cross-sectional CT image

The regions with probability larger than 0.6 [17] on the original NIRS chemogram are considered as lipid-rich plaque. According to this threshold, pie-shaped binary masks originated from catheter center are generated. However, in most occasions the catheter center does not match to the lumen center; hence, translations and rotations are required when co-registering the catheter-centered mask from the IVUS domain to the CTA image domain. The calcium masks were drawn manually by a clinical expert. The registered binary mask labels of lipid-rich and calcified plaques on CTA image are shown in Fig. 2d, e, respectively.

**Fig. 3:**
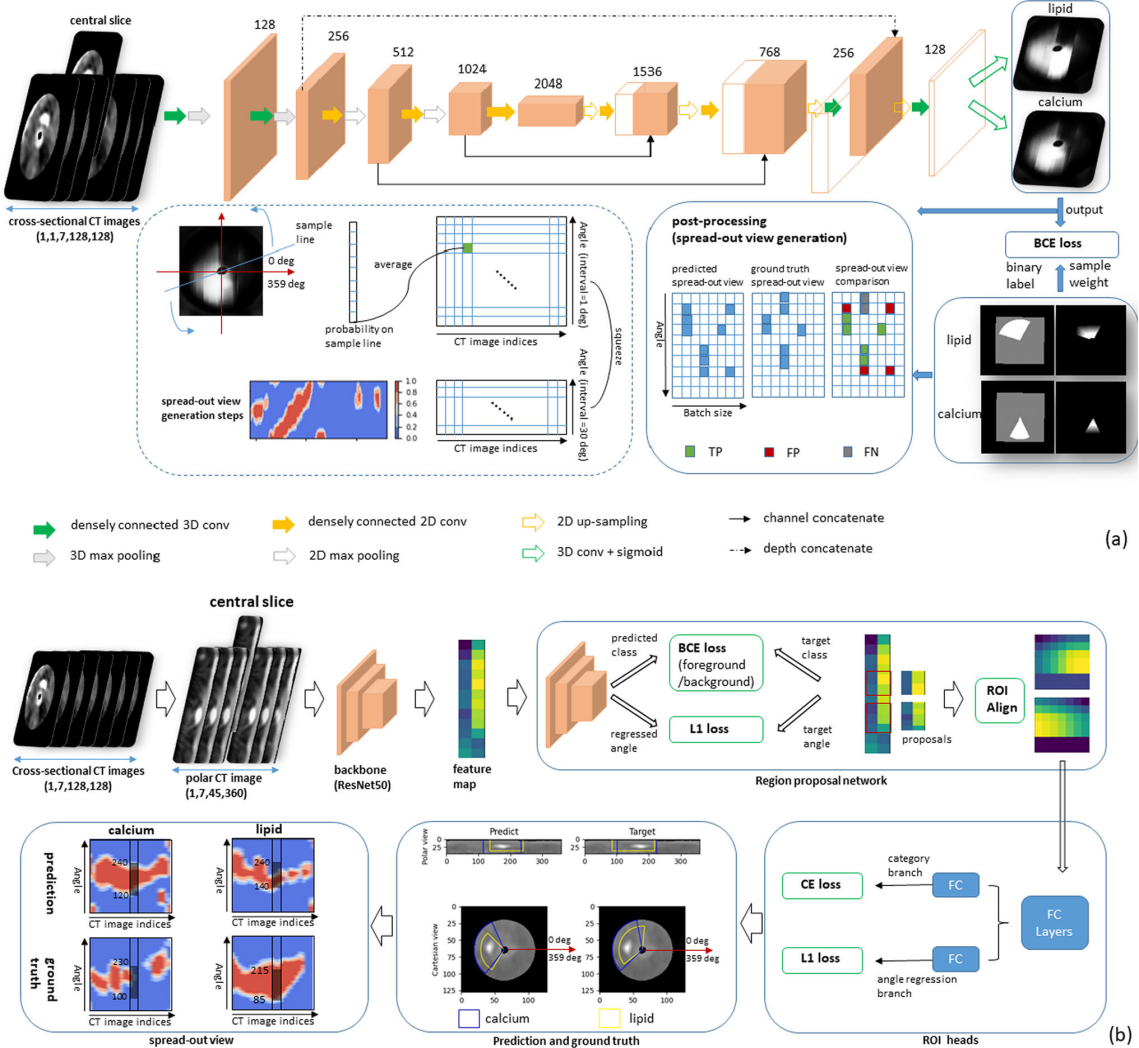
**a** Pipeline of 2.5D Dense U-Net-based method; **b** pipeline of 2.5D Mask R-CNN-based method

Registered masks are transformed to boxes in the polar view to fit the architecture of Mask R-CNN shown in Fig. 3b.

The widely used semi-automated plaque analysis software (QAngioCT Research Edition 3.1, Medis Medical Imaging Systems BV, the Netherlands) [18] was used to segment the CTA data and classify plaque composition in the conventional approach. CTA analysis was performed by an experienced operator with an established reproducibility blindly to the NIRS-IVUS images. We will use conventional method to indicate QAngioCT in the following part.

### Data distribution

After excluding the vessels with metal stents, there are 49 patients (44 for training and 5 for validation) in the training set and 15 patients in the independent test set. In the 2.5D Dense U-Net experiment, 18468 (lipid:1931, calcified:3297, mixed:1979, normal:11261), 2290 (lipid:392, calcified:247, mixed:171, normal:1480) and 7839 (lipid:851, calcified:1240, mixed:670, normal:5078) CTA frames separately from 44, 5 and 15 patients were used for training, validation and testing.

In the 2.5D Mask R-CNN experiment, CTA frames with plaque angle less than 10 degrees were excluded, and CTA frames without lipid or calcified tissue were also excluded in the training. 6640 (lipid:1768, calcified:3019, mixed:1853), 789 (lipid:372, calcified:246, mixed:171) and 7818 (lipid:833, calcified:1237, mixed:667, normal:5081) CTA frames separately from 44, 5 and 15 patients were used for training, validation and testing.

## Methods

The 2.5D Dense U-Net-based method and the 2.5D Mask R-CNN-based method have different advantages in plaque prediction. The cross-sectional CTA images can be used directly as input for the Dense U-Net to learn global features to *distinguish plaques* but with a separate post-processing step shown in Fig. 3a for angular analysis. The Mask R-CNN does not need the post-processing because it is performed in polar view and the angular information can be obtained directly.

### Prediction workflow

#### Dense U-Net

The pipeline of the 2.5D Dense U-Net is shown in Fig. 3a . At the end of the pipeline of the U-Net, the prediction of lipid-rich and calcified plaques is obtained from two individual branches. A pixel-wise gradient sample weight is used to restrict the loss function during the training process. A special post-processing is applied to generate a spread-out plot to evaluate the model performance for the prediction of the plaque distribution.

Weighted binary cross entropy loss is defined in Eq. (1) to optimize separated branches in the 2.5D Dense U-Net. In Eq. (1), *predict* represents the predicted segmentation shown in Fig. 3a; *target* represents the pie-shaped binary label shown in Fig. 2. $weight_{sample}$ represents the pixel-wise gradient sample weight shown in Fig. 2. The pixel value on the sample weight map is inversely proportional to the distance between this pixel and the lumen center. $Reg_{kernel}$ is a $l_2$ norm regularization term which is used as a penalty term of convolutional kernel to avoid overfitting during the training.

1 $$\begin{aligned} \mathrm{{Loss}}_{\mathrm{{DenseUNet}}}= & {} \mathrm{{BCE}}(\mathrm{{predict}},\mathrm{{target}})\times \mathrm{{weight}}_{\mathrm{{sample}}}+\mathrm{{Reg}}_{\mathrm{{kernel}}}\nonumber \\{} & {} \mathrm{{weight}}_{\mathrm{{sample}}}\propto \mathrm{{lumen}}\, \mathrm{{distance}} \end{aligned}$$

#### Mask R-CNN

The pipeline of the 2.5D Mask R-CNN is shown in Fig. 3(b). In this study, we adopt the classical Mask R-CNN architecture with a ResNet50 [19] as a backbone. To take advantage of the context information along the longitudinal direction of the vessels, 2.5D polar CT images are used as the input to the network. The loss is $\mathrm{{loss}}_{\mathrm{{classification}}}+\mathrm{{loss}}_{\mathrm{{regression}}}+\mathrm{{loss}}_{\mathrm{{segmentation}}}$ in the classical Mask R-CNN. There are no specific segmentation labels for plaques. Therefore, the segmentation branch in the classical Mask R-CNN [15] architecture is discarded in our architecture.

The loss function of 2.5D Mask R-CNN is a multi-task loss, combining a binary cross entropy loss with a $l_1$ loss, shown as Eq. (2). $\mathrm{{loss}}_{\mathrm{{cls}}_{\mathrm{{bf}}}}$ and $\mathrm{{loss}}_{\mathrm{{reg}}_{\mathrm{{bf}}} }$ represent the classification loss and angle regression loss for distinguishing foreground and background; $\mathrm{{loss}}_{\mathrm{{cls}}}$ and $\mathrm{{loss}}_{\mathrm{{reg}}}$ represent further classification and regression loss used to separate different kinds of plaques. $\mathrm{{predict}}_{\mathrm{{bf}}}^{\mathrm{{class}}}$, $\mathrm{{predict}}_{\mathrm{{bf}}}^{\mathrm{{angle}}}$, $\mathrm{{predict}}^{\mathrm{{class}}}$ and $\mathrm{{predict}}^{\mathrm{{angle}}}$ are the predicted category and regressed angle. $\mathrm{{target}}_{\mathrm{{bf}}}^{\mathrm{{class}}}$, $\mathrm{{target}}_{\mathrm{{bf}}}^{\mathrm{{angle}}}$, $\mathrm{{target}}^{\mathrm{{class}}}$ and $\mathrm{{target}}^{\mathrm{{angle}}}$ are the target category and target angle.

2 $$\begin{aligned} \mathrm{{Loss}}_{\mathrm{{MaskRCNN}}}= & {} \mathrm{{loss}}_{\mathrm{{cls}}_{\mathrm{{bf}}}}+\mathrm{{loss}}_{\mathrm{{reg}}_{\mathrm{{bf}}}}+\mathrm{{loss}}_{\mathrm{{cls}}}+\mathrm{{loss}}_{\mathrm{{reg}}} \nonumber \\= & {} \mathrm{{BCE}}(\mathrm{{predict}}_{\mathrm{{bf}}}^{\mathrm{{class}}},\mathrm{{target}}_{\mathrm{{bf}}}^{\mathrm{{class}}})\nonumber \\{} & {} +L1(\mathrm{{predict}}_{\mathrm{{bf}}}^{\mathrm{{angle}}},\mathrm{{target}}_{\mathrm{{bf}}}^{\mathrm{{angle}}})\nonumber \\{} & {} +BCE(\mathrm{{predict}}^{\mathrm{{class}}},\mathrm{{target}}^{\mathrm{{class}}}) \nonumber \\{} & {} +L1(\mathrm{{predict}}^{\mathrm{{angle}}},\mathrm{{target}}^{\mathrm{{angle}}}) \end{aligned}$$

### Training of the networks

All the experiments in this study used Adam optimizer with a learning rate of 0.0001 to train 50 epochs with a batch size of 32. The 2.5D data block for the dense U-Net was randomly rotated from 0 to 359 degrees around the image center which could increase the variation in plaque occurring angle. The size of the input data block is 128 $\times$ 128 $\times$ 7 for the 2.5D Dense U-Net and 45 $\times$ 360 $\times$ 7 for the 2.5D Mask R-CNN.

To avoid overfitting, a 50% random dropout was applied to the final layer of the encoder and a $l_2$ norm regularization was introduced to each convolutional layer in the 2.5D Dense U-Net. A data ratio of *lipid* : *calcium* : *mixed* : *normal* tissue was set as 3 : 1 : 1 : 5 per batch.

The masking branch in the classical Mask R-CNN network was discarded since accurate segmentation labels of different plaque types do not exist, and only the branches for classification and regression were maintained. Bounding box initialization was performed just on the angle direction (*x*-axis of the polar image) because no ground truth information along the depth direction (*y*-axis of the polar image) exists.

### Evaluation method

In this patient-level study, a lesion is defined as a certain region in the vessel that contains an elevated $(\ge 40\%)$ plaque burden. In total, 91 lesions were extracted from the total 48 test vessels of 15 patients according to the expert’s knowledge and all the metrics were calculated on the lesion level. *Accuracy* and *F1-score*, calculated from the spread-out view, are used to evaluate the prediction. IOU, Dice and Hausdorff distance, which are commonly used in the pixel-based segmentation, are not applicable in this study. Because these metrics are calculated in the image domain, but there are no pixel-level segmentation labels. The spread-out view shows the plaque distribution on the cross-sectional CTA images and can give a comprehensive overview about the continuity of the plaques in a vessel. The spread-out view generation process is shown on the left-bottom part in Fig. 3a. If the coordinate of one element on the spread-out view is $(\alpha ,n)$, then the value of this element represents the probability that there is a plaque at $\alpha$ degree on CT image *n*.

Violin plots combined with box plots are used to show the distribution of spread-out view-based accuracy and F1-score of the lesions. The spread-out view-based accuracy and F1-score are defined by $\mathrm{{Accuracy}}(\mathrm{{SpreadOut}}_{\mathrm{{pred}}},\mathrm{{SpreadOut}}_{\mathrm{{gt}}})$ and $F1(\mathrm{{SpreadOut}}_{\mathrm{{pred}}},\mathrm{{SpreadOut}}_{\mathrm{{gt}}} )$ that the inputs are predicted spread-out view and ground truth spread-out view derived from NIRS-IVUS estimations.

## Results

### Plaque localization

**Fig. 4:**
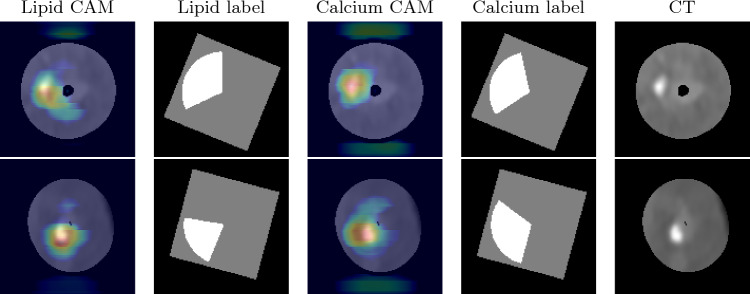
Cross-sectional plaque localization predicted by 2.5D Dense U-Net. From left to right there is the Grad-CAM view of the lipid-rich plaque, pie-shaped label of the lipid-rich plaque, Grad-CAM view of the calcified plaque and pie-shaped label of calcified plaque as well as the CT image from the LCx (1st row) and from the LAD (2nd row)

**Fig. 5:**
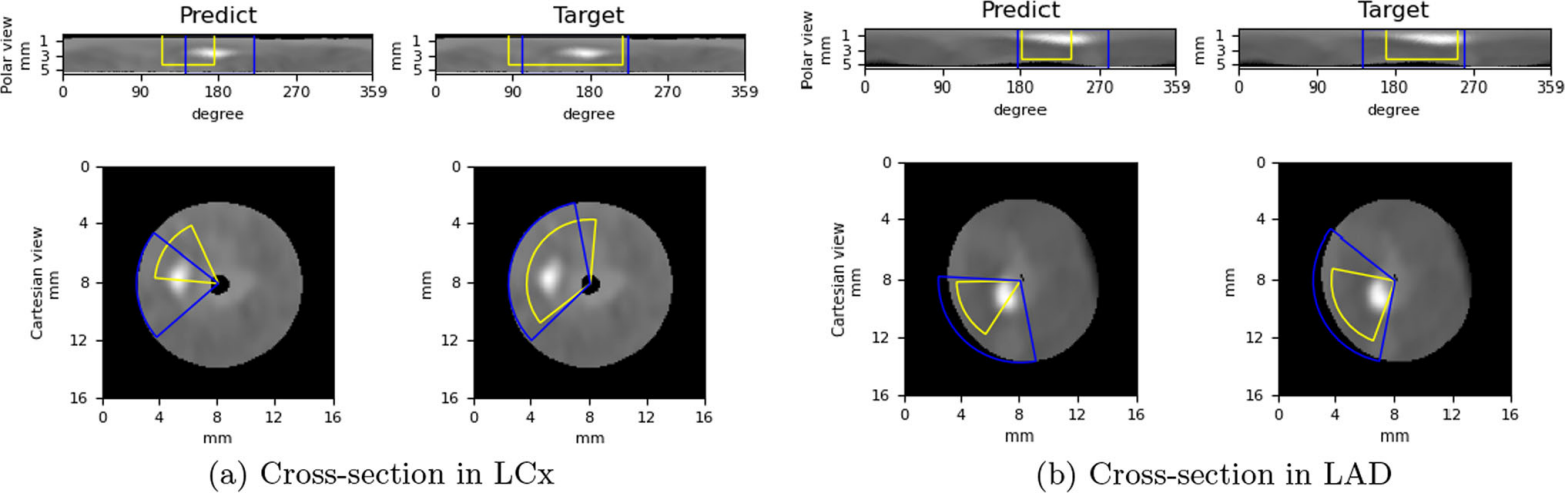
Cross-sectional plaque localization predicted by the 2.5D Mask R-CNN. **a** Predicted and target bounding boxes indicating lipid-rich (yellow) and calcified (blue) plaque position on a cross-section in the LCx; **b** predicted and target bounding boxes indicating lipid-rich (yellow) and calcified (blue) plaque position on a cross section in the LAD. The difference in radius between lipid-rich and calcified plaques is only for the purpose of visualization

The first row and second row in Fig. 4 show the gradient-weighted class activation mapping (Grad-CAM) [20] generated with the 2.5D Dense U-Net predictions and the corresponding pie-shaped label for two cross-sections located in a LCx (left circumflex artery) and a LAD (left anterior descending artery), respectively. Figure 5 shows the bounding boxes generated with the 2.5D Mask R-CNN predictions.

The 2.5D Mask R-CNN uses bounding boxes to localize the plaque regions as shown in Fig. 5. The blue boxes represent calcified plaques, and the yellow boxes represent lipid-rich plaques. We show two different views in Fig. 5, a polar view and the Cartesian view. For the polar view, the *x*-axis shows the angle coordinate from 0 degree to 359 degrees and *y*-axis shows the radius from 0 to 5.625 mm. The plaque detection is performed on the polar CT images, and the predicted rectangular bounding boxes are mapped on the Cartesian view as pie-shaped bounding boxes for a more comprehensive visualization. The *x*-coordinate of left and right side of the rectangular bounding box directly represents the start and end angle; hence, there is no need to perform a post-processing step as for the 2.5D Dense U-Net.

In the following section, we show the spread-out views based on the predicted cross-sectional plaque distributions and use these spread-out views to calculate metrics for the evaluation of the trained model.

### Tissue-type distribution

**Fig. 6:**
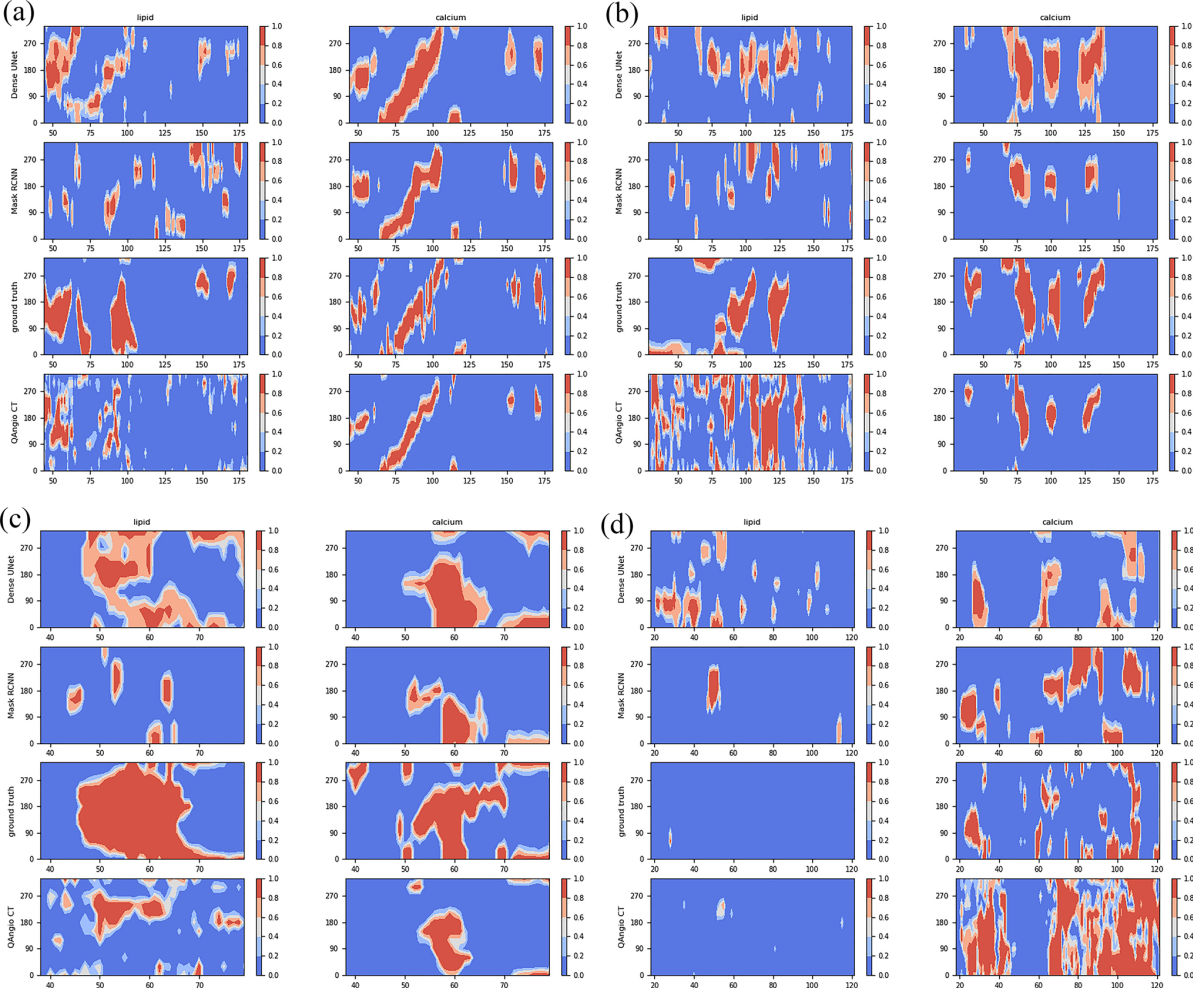
Spread-out view of lipid-rich and calcified plaques of four arteries in the test dataset. The spread-out views were predicted by Dense U-Net, Mask R-CNN, NIRS-IVUS and QAngioCT, respectively. *X*-axis: indices of CT image. *Y*-axis: plaque occurring angle from 0 degree and 359 degree. Color bar: probability of plaque appearance from 0 to 1

Spread-out views of four arteries are shown in Fig. 6. In the 2.5D Dense U-Net experiment, prediction thresholds for both lipid-rich and calcified plaques were 0.6. This means that only predictions with a probability higher than 0.6 are used in the final results. In the 2.5D Mask R-CNN experiment, the threshold of the lipid-rich plaques is 0.3 and that of calcified plaque is 0.5 which means that the predicted bounding boxes with a higher probability are used in the results during the evaluation. These thresholds are empirically determined and a trade-off between spread-out view-based FN and FP. If the threshold is high, the predicted results with moderate probability will be rejected. The FN will increase and the FP will decrease. On the other hand, a low threshold allows more predicted results with low confidence which may underestimate predictions.

In Fig. 6, the two DL-based methods and the conventional method are compared with the NIRS-IVUS estimations treated as a ground truth in the third row. The areas with lipid-rich plaques are scattered on the spread-out views from the Mask R-CNN. The lipid-rich areas between neighboring CTA images on the spread-out views predicted by the Dense U-Net are more continuous than both the Mask R-CNN and the conventional methods.

The lumen intensity of the vessel shown in Fig. 6d is higher than the others. Therefore, the threshold (350 HU) used to distinguish calcified plaques from the lumen area in the conventional method is invalid and causing meaningless predictions at the bottom-right spread-out view in Fig. 6d. The areas with calcified plaques are visually similar among the predicted spread-out views, ground truth and the conventional method, except the high lumen intensity case shown in Fig. 6d.

### Prediction evaluation

**Fig. 7:**
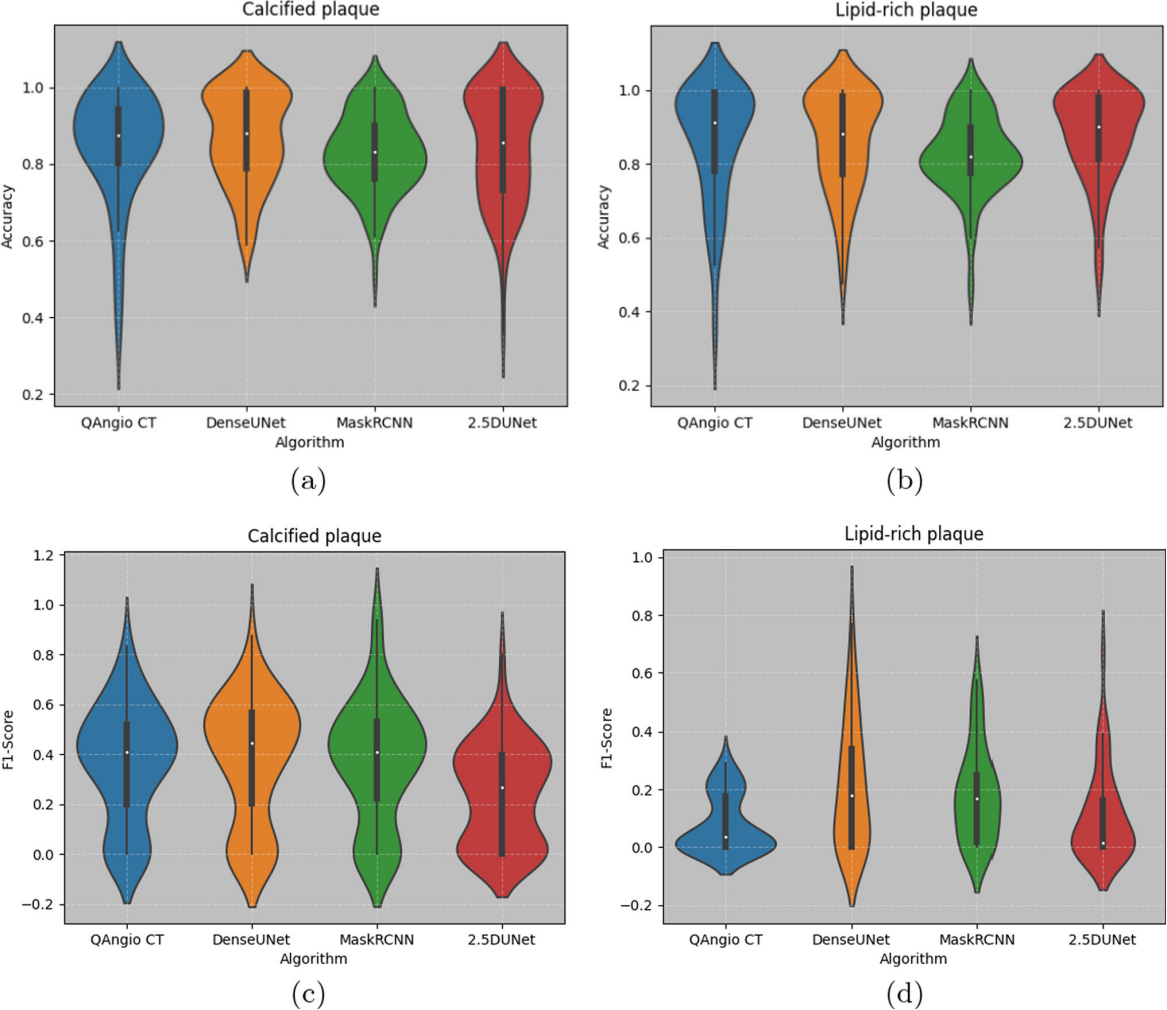
Distribution of the spread-out view-based accuracy and F1-scores for 91 lesions. Blue, yellow, green and red violin plots represent the distribution of the conventional method, 2.5D Dense U-Net, 2.5D Mask R-CNN and classical 2.5D U-Net

To evaluate the similarity between predicted spread-out views and ground truth spread-out views numerically, the accuracy and F1-score were calculated on the spread-out views of the calcified and lipid-rich plaques. The results are shown in Fig. 7. The blue, yellow, green and red violin plots represent the different methods used for plaque prediction, QAngioCT, 2.5D Dense U-Net, 2.5D Mask R-CNN and the classical 2.5D U-Net.

The median value of the *Accuracy* of the *calcified* plaques in the box plot of Fig. 7a is similar among different methods. The distribution of values in the Dense U-Net and Mask R-CNN is more aggregated than the conventional method and classical 2.5D U-Net.

The Dense U-Net shows the highest median value in the *F1-score* distribution of calcified plaques shown in Fig. 7c. The median of the conventional method and Mask R-CNN is comparable to each other but higher than classical 2.5D U-Net.

Moreover, the Dense U-Net performs better in both median and density plots when comparing the Accuracy metric distribution of lipid plaques as shown in Fig. 7b.

The median value of the F1-score is comparable between the Dense U-Net and Mask R-CNN estimations for the lipid plaques, and both are around 20% in Fig. 7d. The Mask R-CNN has a more aggregated distribution.

The numerical evaluation, median and mean value of Accuracy and F1-score, are shown in Tables 1 and 2 for the lipid-rich and calcified plaques, respectively, in 91 lesions.

For the prediction of lipid-rich plaques in Table 1, the 2.5D Dense U-Net had the highest median (0.18) and mean (0.21) value for the F1-score among all studied methods. The performance of the 2.5D Mask R-CNN was lower with a median and mean value of 0.17 and 0.18, respectively. The F1-score of both methods proposed in this paper exceeded the score of the conventional method and classical 2.5 U-Net, with their median and mean values being around 6 and 3 times higher than those of the conventional method.

In Table 2, the difference between the mean F1-score of the 2.5D Dense U-Net and the conventional method was only 0.01, but the median values of the F1-score for the 2.5D Dense U-Net were 0.03 higher than that of the conventional method. The results of the 2.5D Mask R-CNN were lower than the 2.5D Dense U-Net but comparable with the conventional method. The median of the F1-score is still 0.14 higher than the classical 2.5D U-Net. Compared to the ground truth, the characterization of calcified plaque of the conventional method is accurate except for the cases with high intensity lumen (HU>350) shown in Fig. 6d.

## Discussion and conclusion

Two different methods for plaque angle prediction on cross-sectional CT images are proposed. We first propose to use NIRS-IVUS plaque labels to train the network to predict the cross-sectional distribution of lipid and calcified plaques in the coronary CTA arteries. It is an angle-level multi-target classification instead of an image-level binary classification in this study.

The 2.5D Mask R-CNN pipeline has fewer steps than the 2.5D Dense U-Net, but the metrics of the predicted results shown in Tables 1 and 2 indicate that Dense U-Net has a slight advantage for the prediction of both lipid-rich plaques and calcified plaques.

Except for the high intensity lumen case shown in Fig. 6d, the conventional method can generate confident calcium spread-out views based on fixed HU thresholds (350 HU for calcified plaques). Small calcified regions cannot be detected if the intensity of the lumen is higher.

The calcified areas with a high probability are clustered on the calcium spots in Fig. 4. The Dense U-Net-based method tends to over-estimate the angle range of the calcium plaques compared to the Mask R-CNN as shown in Fig. 6a.

The comparison between the different methods indicates that the proposed methods have capabilities to improve the detection performance of lipid-rich plaques. The median values of the F1-score of the proposed methods are 6 times higher than that of a conventional method which needs the EEM detection.

**Table 1:** Median and average of spread-out view-based accuracy and F1-score of lipid-rich plaque predictions in 91 lesions

Methods	Accuracy	F1-score
	median(mean)	median(mean)
2.5D Dense U-Net (with grad-weight)$*$	**0.88 (0.86)**	**0.18 (0.21)**
2.5D Mask R-CNN (angle regression)$*$	**0.82 (0.82)**	**0.17 (0.18)**
2.5D U-Net [13] (with grad-weight)	0.90 (0.88)	0.02 (0.11)
QAngioCT^†^ [18]	0.91 (0.86)	0.03 (0.08)

The metrics of our proposed pipeline are in bold type

$*$ Represents the proposed pipeline; $\dagger$Represents the conventional method)

**Table 2:** Median and average of spread-out view-based accuracy and F1-score of calcified plaque predictions in 91 lesions

Methods	Accuracy	F1-score
	median(mean)	median(mean)
2.5D Dense U-Net (with grad-weight)$*$	**0.88 (0.87)**	**0.44 (0.38)**
2.5D Mask R-CNN (angle regression)$*$	**0.83 (0.83)**	**0.41 (0.37)**
2.5D U-Net [13] (with grad-weight)	0.86 (0.84)	0.27 (0.24)
QAngioCT^†^ [18]	0.88 (0.85)	0.41 (0.37)

The metrics of our proposed pipeline are in bold type

$*$ Represents the proposed pipeline; $\dagger$Represents the conventional method

The high confidence areas of lipid-rich plaques are distributed around calcium spots. The difference between the Grad-CAM of the lipid-rich plaques and calcified plaques demonstrates that although the predicted plaque regions overlap, the focal areas of the network are different for different kinds of plaques.

The results in this paper suggest that it is promising to distinguish different kinds of plaques on CT images based on deep learning networks. However, it is uncertain whether all the lipid-rich areas can be detected on the cross-sectional CTA images using DL methods. The predictions of calcified plaques by the 2.5D Dense U-Net resemble the ground truth, which indicates that the registrations are accurate to guide the network to extract useful features. Moreover, comparing the violin plots that belong to the different methods, it is clear that the Dense U-Net and Mask R-CNN-based predictions of calcified plaques are similar or better than a conventional method.

The main limitation in this paper is that the dataset used for training is relative small; only 176 arteries from 64 patients are involved in this study. Another limitation is that some areas with lipid-rich tissue are hard to distinguish from surrounding tissues and normal vessel wall. Furthermore, the resolution of CT images is relatively low compared to NIRS-IVUS and blooming artifacts caused by calcified plaques make it hard to distinguish calcified plaques from the contrast in the lumen. The next-generation CT scanners such as photon-counting CT may overcome these limitations by its higher resolution and stronger artifact suppression.

In conclusion, this study presents two methods to predict lipid-rich and calcified plaques on the cross-sectional CT images using DL approaches. To the best of our knowledge, this is the first study to explore the mapping relationship between lipid-rich plaques on CT images and that on NIRS-IVUS images. Though the plaque detection is a challenging topic based on the currently used CT technology, the two methods proposed in this paper contribute to finer predictions of lipid-rich and calcified plaques. Especially for the lipid-rich plaques, the metrics calculated on the spread-out views are improved compared to the conventional method.

## References

[1] Bertrand M-J, Lavoie-L’Allier P, Tardif J-C (2017). Near-infrared spectroscopy (NIRS): a novel tool for intravascular coronary imaging. Dev Near-Infrared Spectrosc.

[2] Saremi F, Achenbach S (2015). Coronary plaque characterization using CT. Am J Roentgenol.

[3] Gudigar A, Nayak S, Samanth J, Raghavendra U, Ashwal AJ, Barua PD, Hasan MN, Ciaccio EJ, Tan R-S, Rajendra Acharya U (2021). Recent trends in artificial intelligence-assisted coronary atherosclerotic plaque characterization. Int J Environ Res Public Health.

[4] Finn AV, Nakano M, Narula J, Kolodgie FD, Virmani R (2010). Concept of vulnerable/unstable plaque. Arterioscler Thromb Vasc Biol.

[5] Sathyanarayana S, Carlier S, Li W, Thomas L (2009). Characterisation of atherosclerotic plaque by spectral similarity of radiofrequency intravascular ultrasound signals. EuroIntervention.

[6] Tearney GJ, Yabushita H, Houser SL, Aretz HT, Jang I-K, Schlendorf KH, Kauffman CR, Shishkov M, Halpern EF, Bouma BE (2003). Quantification of macrophage content in atherosclerotic plaques by optical coherence tomography. Circulation.

[7] Schuurman A-S, Vroegindewey M, Kardys I, Oemrawsingh RM, Cheng JM, Boer S, Garcia-Garcia HM, Geuns R-J, Regar ES, Daemen J, Mieghem NM, Serruys PW, Boersma E, Akkerhuis KM (2018). Near-infrared spectroscopy-derived lipid core burden index predicts adverse cardiovascular outcome in patients with coronary artery disease during long-term follow-up. Eur Heart J.

[8] Liu H, Wingert A, Wang J, Zhang J, Wang X, Sun J, Chen F, Khalid SG, Jiang J, Zheng D (2021). Extraction of coronary atherosclerotic plaques from computed tomography imaging: a review of recent methods. Front Cardiovasc Med.

[9] Daghem M, Bing R, Fayad ZA, Dweck MR (2020). Noninvasive imaging to assess atherosclerotic plaque composition and disease activity: coronary and carotid applications. JACC Cardiovasc Imaging.

[10] Szilveszter B, Celeng C, Maurovich-Horvat P (2016). Plaque assessment by coronary CT. Int J Cardiovasc Imaging.

[11] Zreik M, Hamersvelt RW, Wolterink JM, Leiner T, Viergever MA, Išgum I (2019). A recurrent CNN for automatic detection and classification of coronary artery plaque and stenosis in coronary CT angiography. IEEE Trans Med Imaging.

[12] Liu J, Jin C, Feng J, Du Y, Lu J, Zhou J (2019) A vessel-focused 3D convolutional network for automatic segmentation and classification of coronary artery plaques in cardiac CTA. In: Statistical Atlases and Computational Models of the Heart. Atrial Segmentation and LV Quantification Challenges, vol. 11395, (pp. 131–141). 10.1007/978-3-030-12029-0_15

[13] Vu MH, Grimbergen G, Nyholm T, Löfstedt T (2020). Evaluation of multislice inputs to convolutional neural networks for medical image segmentation. Med Phys.

[14] Huang G, Liu Z, Van Der Maaten L, Weinberger KQ (2017) Densely connected convolutional networks. In: 2017 IEEE Conference on Computer Vision and Pattern Recognition (CVPR), (pp. 2261–2269). 10.1109/CVPR.2017.243

[15] He K, Gkioxari G, Dollár P, Girshick R (2017) Mask R-CNN. In: 2017 IEEE International Conference on Computer Vision (ICCV), (pp. 2980–2988). 10.1109/ICCV.2017.322

[16] De Graaf MA, Broersen A, Kitslaar PH, Roos CJ, Dijkstra J, Lelieveldt BP, Jukema JW, Schalij MJ, Delgado V, Bax JJ, Johan HCR, Arthur JS (2013). Automatic quantification and characterization of coronary atherosclerosis with computed tomography coronary angiography: cross-correlation with intravascular ultrasound virtual histology. Int J Cardiovasc Imaging.

[17] O’Brien A, LaCombe A, Stickland A, Madder RD (2016) Intracoronary near-infrared spectroscopy: an overview of the technology, histologic validation, and clinical applications. Global Cardiol Sci Pract. 10.21542/gcsp.2016.18 10.21542/gcsp.2016.18 PMC5642785 29043266

[18] Park H-B, Lee BK, Shin S, Heo R, Arsanjani R, Kitslaar PH, Broersen A, Dijkstra J, Ahn SG, Min JK, Chang H-J, Hong M-K, Jang Y, Chung N (2015). Clinical feasibility of 3D automated coronary atherosclerotic plaque quantification algorithm on coronary computed tomography angiography: comparison with intravascular ultrasound. Eur Radiol.

[19] He K, Zhang X, Ren S, Sun J (2016) Deep residual learning for image recognition. In: 2016 IEEE Conference on Computer Vision and Pattern Recognition (CVPR), (pp. 770–778). 10.1109/CVPR.2016.90

[20] Selvaraju RR, Cogswell M, Das A, Vedantam R, Parikh D, Batra D (2017) Grad-CAM: visual explanations from deep networks via gradient-based localization. In: 2017 IEEE International Conference on Computer Vision (ICCV), (pp. 618–626). 10.1109/ICCV.2017.74

